# MAPI: a software framework for distributed biomedical applications

**DOI:** 10.1186/2041-1480-4-4

**Published:** 2013-01-11

**Authors:** Johan Karlsson, Oswaldo Trelles

**Affiliations:** 1 Computer Architecture Department, University of Málaga, Complejo Tecnológico, Campus de Teatinos, Málaga, 29080, Spain

**Keywords:** Service-oriented architectures, Web-service integration, Software framework

## Abstract

**Background:**

The amount of web-based resources (databases, tools etc.) in biomedicine has increased, but the integrated usage of those resources is complex due to differences in access protocols and data formats. However, distributed data processing is becoming inevitable in several domains, in particular in biomedicine, where researchers face rapidly increasing data sizes. This big data is difficult to process locally because of the large processing, memory and storage capacity required.

**Results:**

This manuscript describes a framework, called MAPI, which provides a uniform representation of resources available over the Internet, in particular for Web Services. The framework enhances their interoperability and collaborative use by enabling a uniform and remote access. The framework functionality is organized in modules that can be combined and configured in different ways to fulfil concrete development requirements.

**Conclusions:**

The framework has been tested in the biomedical application domain where it has been a base for developing several clients that are able to integrate different web resources. The MAPI binaries and documentation are freely available at http://www.bitlab-es.com/mapi under the Creative Commons Attribution-No Derivative Works 2.5 Spain License. The MAPI source code is available by request (GPL v3 license).

## Background

The World Wide Web (WWW) has emerged as a gallery of resources, such as Web Services (WS) and datasets, which can be discovered, combined and exploited to enhance our capacity of producing new knowledge. One prominent example is the BioCatalogue [1] repository with metadata describing over 2200 WS (November 2011).

The potential of using WS to support biomedical research has been widely reported. For example, in [2,3], WS are used to establish genome-disease associations which are necessary for patient genome sequencing to support determination of diagnosis or therapy. In [4], semantics are used to enrich the patient record system, in particular for tasks related to drug prescription (drug interactions, medical insurance coverage for the drug etc.). The authors show how web-services can be used to communicate information between the legacy systems and databases.

However, sending the output from a WS to another WS (i.e. as workflows or pipelines) is complex because of differences in WS communication protocols (varying from SOAP [5] to WS using REST [6] principles) and data formats (for example FASTA [7], GenBank [8] and FASTQ [9]). WS metadata describing tool inputs and outputs and syntax description of formats (datatypes) in shared repositories simplifies the development of user-friendly client software that can combine WS as workflows [10]. With such metadata, it is also possible to apply tools such as ReadSeq [11] to automatically transform biological sequence data between formats.

This paper describes a software framework (MAPI) which provides support for WS integration. MAPI addresses the following aspects of WS integration and usage:

· Management and discovery of WS instances in metadata registries

· Unification of WS metadata

· WS invocation (execution) and data format conversion.

In [12] we showed how MAPI facilitates client development by allowing the developers to focus on GUI aspects. This paper gives complementary background and details, in particular with respect to the metadata schema in MAPI (see Section “Common (shared) model”) and aspects related to addressing heterogeneity in WS and user data (see Section “Seamless data format transformation”). Additionally, this paper exemplifies the usage of MAPI functions for a simple use case (see Section “Use Case– Homologous Protein Finder”) and discusses the role of MAPI in biomedical settings where there are strong requirements for security (see Section “Potential of using WS in biomedicine”).

We will discuss the role of MAPI in the biomedicine domain where a wide variety of formats, protocols and tools are used [13]. As a proof of concept, MAPI provides support for BioMOBY WS [14], WSDL – described SOAP WS (for example, from European Bioinformatics Institute, EBI [15] and DNA Data Bank of Japan, DDBJ [16]), Taverna [17] workflows, WS from INB [18] and ACGT [19] projects.

## Implementation

This section gives an overview of the MAPI software framework and its novel characteristics. The MAPI framework covers functionality related to service-oriented architectures, in particular management of metadata for WS, datatypes, data, files and users. We will describe the modules (components) and their overall functionality.

### BioMOBY datatype taxonomy

It is quite common that the results from one WS invocation must be further analyzed using other web-services. The standard BioMOBY [14] aims to simplify this task by defining a shared datatype taxonomy and a standardized web-service protocol. The taxonomy follows the object-oriented paradigm where data types are related to other data types. Data types can inherit parts from another data type and add additional structure/attributes by including (containing) or consisting of arrays of other data types. For example, the datatype GenericSequence from the datatype taxonomy of BioMOBY MobyCentral inherits from VirtualSequence the attributes id (String) and namespace (String) and length (Integer). GenericSequence adds a new attribute called SequenceString (String) which contains the actual sequence characters.

### Modules

**Figure 1:**
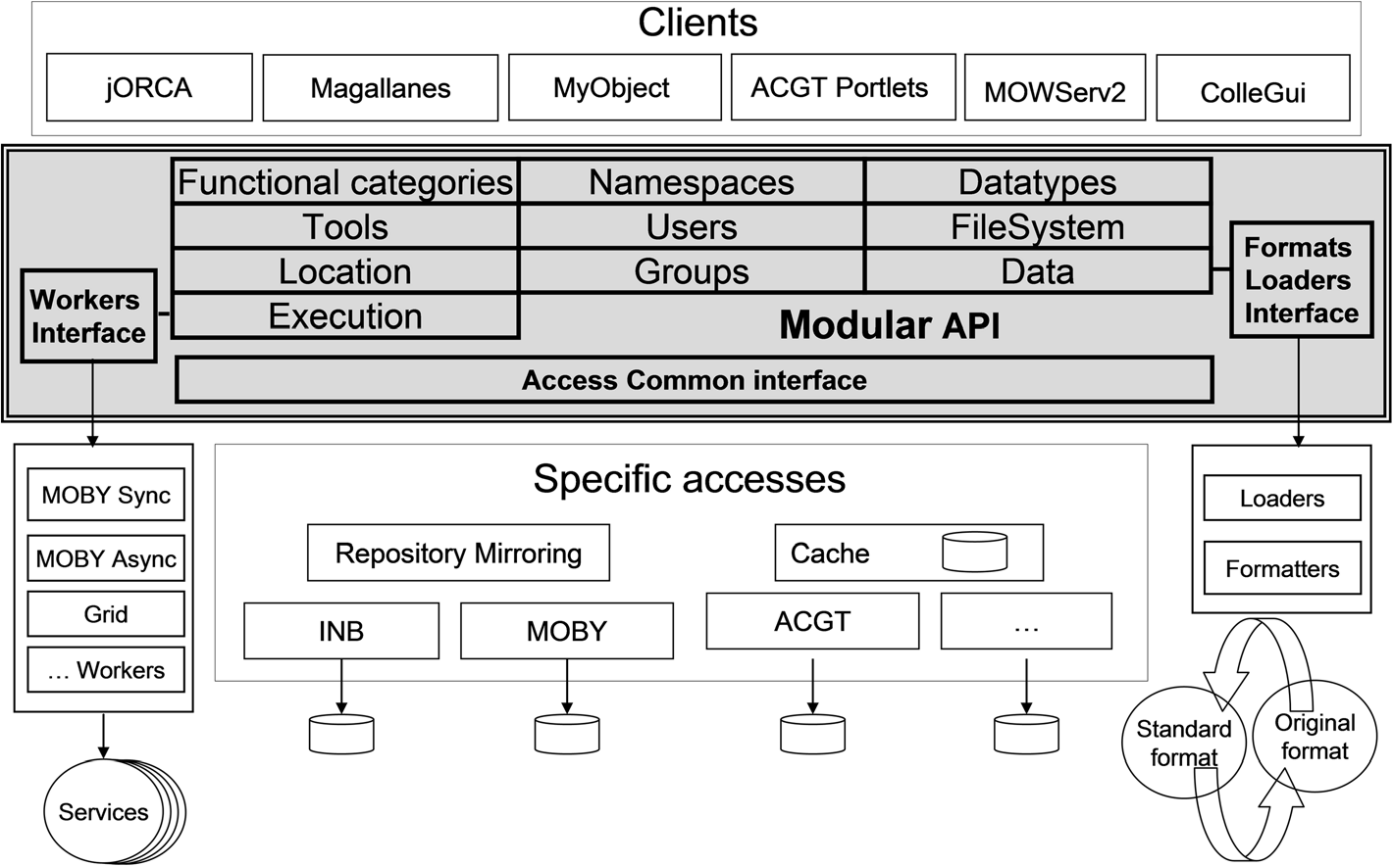
**MAPI architecture. **The figure shows the different software components which comprise the overall framework. Each module has one or several accesses. The Workers, Formatters and Loaders enable the Execution and Data modules to invoke services following different service protocols and data formats respectively.

MAPI modules and their metadata schemas are based on concepts related to WS (e.g., tool, datatype, endpoint, parameter, etc.) and relationships between WS concepts (e.g., the datatype of a parameter). For details about the schemas, please see Figure 1 and Additional file 1: Supplementary material (“Internal data models”).

The following modules are available:

• *Tool*: A tool is an abstract grouping of software components used to solve a specific type of problem. Several types of tools are supported in this module; examples include locally available applications (on a client machine), remotely accessible WS or even complex workflows. Each tool has one or more operations. Each operation has one or more input/output parameters. Each parameter is associated with a specific datatype.

• *ToolLocation*: Each Tool instance can have one or many ToolLocation instances representing service endpoints (mirrors). Note that the instances can specify different WS communication protocols (for example BioMOBY or SOAP) for the same abstract Tool. Multiple endpoints help to create robust and fault-tolerant clients because it is possible to call another endpoint when one or more are not available. The module also provides access to information about the host machine of the endpoint (such as memory, bandwidth etc.).

• *Datatype*: This module manages the shared taxonomy of datatypes. Using such taxonomy is essential for WS interoperability because it obligates WS providers to adhere to the taxonomy. By declaring that a WS works with a specific datatype in the ontology, WS providers guarantee that the WS is able to process data of a specific datatype (or compatible datatypes based on inheritance as declared in the taxonomy). MAPI has taken the approach used in BioMOBY a as base for the DataType module: inheritance (IS relation) and HAS and HASA relations.

• *Functional categories*: This module organizes functional categories in taxonomy. A functional category is a keyword with semantic properties that can be hierarchically arranged. The arrangement is structured in such a way that each resource can be annotated with one or more keywords, from descriptions that range from very specific to generic.

• *Namespaces*: The namespace module stores information about data provenance (data sources). Namespaces provide a method to place resources in context by qualifying elements and attribute names.

• *Data*: This module deals with the management of structured data. Internally, the module transforms user data from different data formats to a common, structured data format (as defined in the datatype module). Clients can programmatically extract different parts of the structured data (using components called Loaders) and/or export data to different formats (using components called Formatters).

• *File System*: This module provides an abstract view of files and folders and permits client software to read and write files/directories regardless of where or how they are physically stored.

• *Execution*: This module provides mechanisms to invoke the tools defined in the Tool and ToolLocation modules. The set of supported tools can be extended by independent plug-ins called Workers and are in charge of actually invoking the tools.

• *Statistics*: This module is used to record and provide statistics about tool usage. This information can be used to analyze the behavior of endpoints (mirrors), identify which endpoints is most frequently used etc. in order to implement more efficient scheduling algorithms.

• *Users*: This module provides the functionality required for handling information about persons (users, data owners or tool providers) and institutions associated with resources. The main strength of this module is its ability to combine with any of the others modules to produce ‘secure versions’, where the access to information (read/write) is restricted based on user rights.

### Functionality

The functionality of each module has been designed around the resource it manages (e.g., users, files, tools, data types). Each module provides methods for accessing, querying and editing metadata. The main functionalities of the modules are:

1. *Retrieval of Resources/Information* (all modules): metadata for a specific resource or all resources (lists) can be retrieved.

2. *Filtering* (all modules): all lists of resources retrieved by the modules can additionally be filtered so that the resources satisfy different criteria (extendable by writing new filters).

3. *Hierarchical Browsing* (File system and Functional categories): for modules that handle resources organized in a taxonomy, the framework provides the functions needed to browse the resources as a tree and change the parent/child relations of the resources (i.e. modifying the taxonomy).

4. *General Editing* (all the modules): every module has methods for adding new resources. In the same way, resources can be deleted and it is possible to configure whether dependent resources will also be deleted in a cascade fashion or whether deletion will be rejected while the dependences exist. Finally, the values of resource attributes can be modified.

5. *Compatibility Search* (Tools, Data and Datatypes): the framework provides functions for finding compatible WS based on the parameter datatypes.

6. *Data Formatting* (Data): the data module has functions for managing the formatters available in the system and converting user data between different formats.

7. *Task Invocation* (Execution): The Execution module manages sub-components (workers) which are able to execute/invoke different types of tools (WS, workflows etc.).

8. *Task Querying* (Execution): This module lets software developers query status and obtain results/statistics from service executions.

A detailed list of the functionality of each module is available at http://www.bitlab-es.com/mapi/.

### Model characteristics

This section describes the main characteristics of the MAPI framework. The design of MAPI has been focused on providing a common and generic model of WS metadata, with the minimal set of metadata necessary to construct client software.

### A flexible modular model

Models for different aspects of WS metadata are separated in different modules and can be combined to adapt to a specific requirement. Because some modules require information from another module, the modules are not totally independent. For example, the Tool module requires the Datatype module to provide the definition of data types used for the parameters of tools defined in the Tool module.

Each module has two layers: Access and Interface. The Access layer is in charge of mapping the information from the data model used in the source repository to the model used in MAPI, while the Interface defines the protocol and programmatic method used by clients to access the functionality of the module (i.e. the public API).

Communication between these two layers is carried out using a common, internal interface.

In addition to the two main layers, it is possible to add more layers (following the same internal interface) to supply new features, such as a cache. Adding more layers does not affect the Interface layer (for example, software clients only need to update the configuration to enable caching).

**Figure 2:**
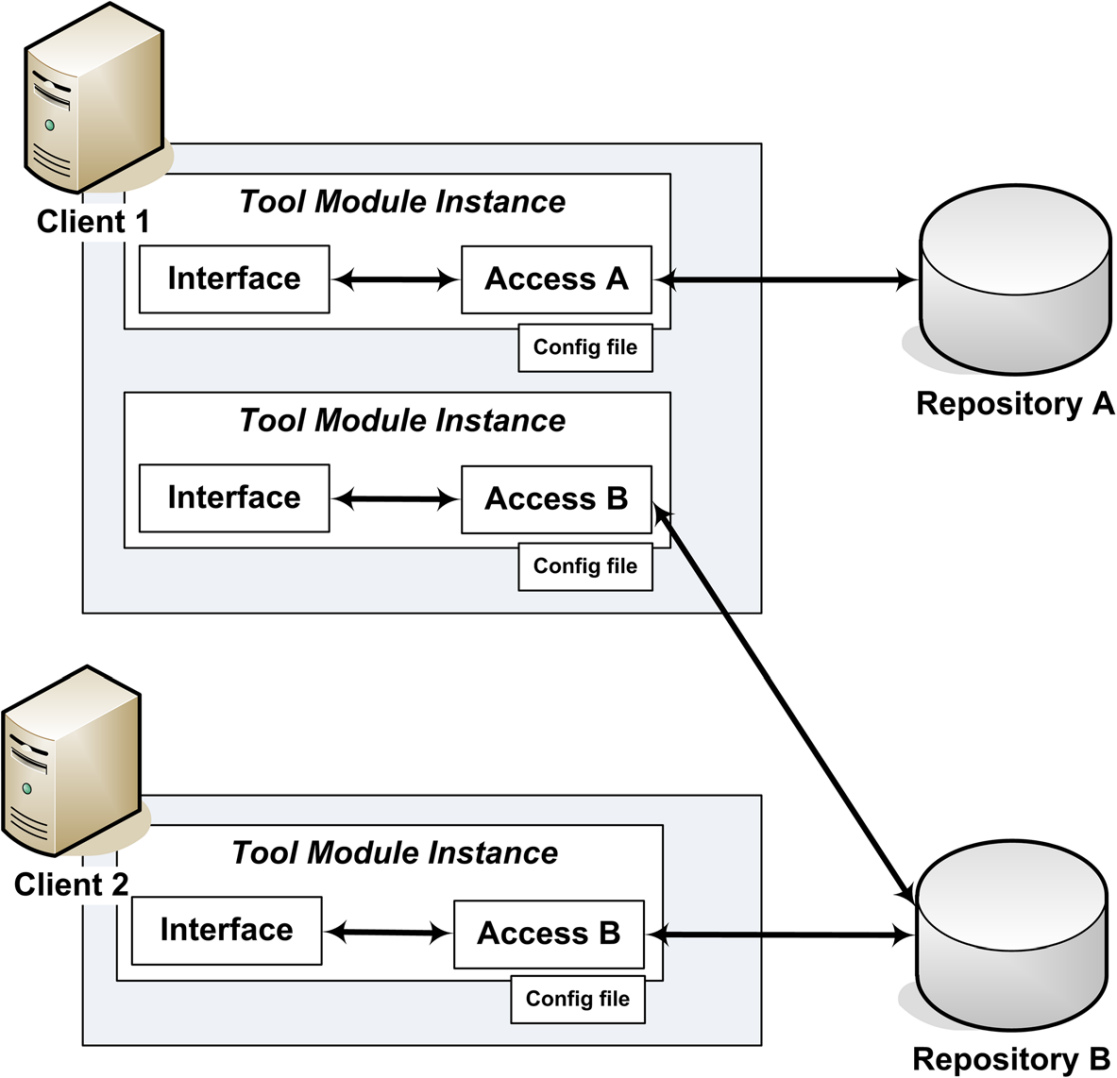
**Setup for two clients. **Two clients have instantiated MAPI modules. Client 1 has two instances of the Tool Module, one where the Interface layer communicates with repository A through Access A and another where it communicates with repository B through Access B. Client 2 only has one instance which communicates to Repository B through Access B. Note that the interface for both clients is always the same, regardless whether they are communicating with repository A or B. The Access B code is also the same for both clients (i.e. the access needs to be developed only once). The specific configuration in each client is controlled through the configuration file. Note that it is necessary to use more modules than only the Tool Module (which needs, at least, the ToolLocation, DataType and FunctionalCategory modules). However, for simplification we only show instances of the Tool Module.

This separation in layers allows flexible clients to be developed. This can be seen in Figure 2, where two different clients share the same access code but are configured to use a different set of tools.

### Common (shared) model

By allowing software developers to work with a shared and common model of WS metadata, it becomes much simpler to construct client software which is able to work with different types of WS because the complexity and heterogeneity in service-oriented architectures are dealt with by MAPI, not by the client software code directly.

Configurations for several taxonomies are supplied with the standard distribution of MAPI (for example, the main BioMOBY MobyCentral taxonomy http://www.biomoby.org or the INB taxonomy http://www.inab.org). However, please note that MAPI is not limited to using a specific datatype or functional category taxonomy since writing new configurations can extend the set of taxonomies available for MAPI.

Similar conditions apply to functional categories. In itself, MAPI does not specify any functional categories but can be configured to use external sources, such as those found in BioMOBY MobyCentral. For example, a user interested in finding services performing a certain task could browse the service tree using a graphical tool which, in turn, uses MAPI functions getFunctionalCategoryRoots to obtain the roots of the taxonomy and recursively call the getChildren and getTools methods for the FunctionalCategory instance to obtain the functional category instances (children) which inherit from the instance and the tools annotated with the instance respectively. Client software uses the same API calls to obtain this information, regardless of which service catalogue is used. MAPI will use the relevant access depending on the configuration.

### Extensibility of data model

Naturally, it is not realistic to establish a data model which successfully predicts all future requirements and WS standards. Therefore, the data model in MAPI is extensible. Additional modules can be implemented for new concepts without affecting existing modules. If some feature is necessary for an existing module, it is possible to extend the existing module (with a new module) and add the new feature (i.e. feature inheritance between modules).

### Seamless data format transformation

As has been mentioned earlier, biomedicine is an example of a research field where a multitude of tools produce and consume many different data formats. One example of such dispersion is a multitude of formats for biological sequence data. This dispersion limits the feasibility of interconnecting WS which require or produce data in different sequence formats.

The data module in MAPI represents structured user data with methods to read, navigate and modify the data structure (this can be compared to APIs to navigate and modify XML DOM trees).

Modifications to the data structure can be applied automatically with two types of MAPI components, Loaders and Formatters. Both types of components are applied based on the datatype taxonomy (inheritance) without user intervention. Components configured to work on a specific parent datatype will also be applied to any data of child datatypes.

Loaders are able to modify the data structure (as defined in the datatype module) of user data. This enables data to be represented in a generic way, but still be compatible with requirements of particular data standards. For example, in BioMOBY, sequences contain several fields besides the actual sequence data: every data object in BioMOBY must have two attributes (identifier and namespace), and every sequence must also contain explicitly the length of the sequence data. When MAPI loads raw sequence data and is asked to produce this data in the BioMOBY format, two loaders are applied seamlessly (without the need for programmers to specify this) to include the required attributes and calculate/add the length of the sequence data. The loader component which adds the two required attributes is always invoked for all BioMOBY data (it is configured to be applied to any data which inherits from the base class; in essence all BioMOBY data). It is possible to develop new loaders and to configure when they are used.

Formatters, also extensible and configurable, are responsible for the serialization of the generic data structure to the actual data format (for example, BioMOBY data is serialized as XML).

Efforts to provide mechanisms for data format transformation exist (see for example [11]), but the approach in MAPI is – to our knowledge – unique in the sense that software developers can specify a set of formatters and loaders (in essence a set of rules) which are applied seamlessly when connecting services.

## Results and discussion

This section will discuss some aspects of the design and implementation of MAPI. The first part provides a case study and gives an overview of the features provided by other systems in comparison with MAPI. The second part comments on different types of service and data heterogeneity, and the mechanisms that MAPI provides to address this.

### Clients implemented using MAPI

MAPI is a software framework aiming to simplify WS integration and client development. The usefulness of MAPI in practice for biomedicine is therefore best represented in higher-level clients implemented using MAPI. In [12], we showed how the MAPI framework can be used to build complex clients. One notable example is jORCA [20] which uses MAPI in different ways: for example the Datatypes, Namespaces and WS trees are built using the DataTypes, Namespaces, Tools and FunctionalCategory modules. In this sub-section, please refer to Table 1 for functionalities referenced. For each module, jORCA asks for the list of tools, datatypes and namespaces (functionality 1) and for relations with the FunctionalCategories (functionality 3). jORCA also makes use of the Filtering functionality (functionality 2) for the quick-search tool; and using the ToolModule, jORCA is able to quickly retrieve the list of compatible tools for a selected datatype (functionality 5). The Execution module along with the ToolLocation module is essential to execute and monitor tools in a transparent way to the final user (functionality 7 and 8).

**Table 1:** Features available in different systems. Legend: [✓] Supported; [NA] Not Available; [L] Limited; [OG] On-Going

**Functionality**	**MAPI**	**BioMOBY**	**Globus**	**UDDI**	**Feta**	**WSMX**	**SADI**
1. Retrieval resources	✓	✓	✓	✓	✓	✓	✓
2. Querying	✓	✓	✓	✓	✓	✓	✓
3. Filtering	✓	L	✓	NA	NA	✓	✓
4. Compatibility search	✓	✓	NA	NA	✓	✓	✓
5. Retrieval information	✓	✓	✓	✓	✓	✓	✓
6. Browsing tree	✓	L	NA	NA	NA	L	NA
7. Data Formatting	✓	NA	NA	NA	NA	✓	NA
8. Task invocation	✓	✓	✓	NA	NA	✓	NA
9. Task query	✓	✓	NA	NA	NA	✓	NA
10. Task scheduling	OG	NA	✓	NA	NA	L	NA
11. Adding resources	✓	✓	✓	✓	✓	✓	✓
12. Delete resources	✓	L	✓	✓	✓	✓	✓
13. General aditing	✓	NA	NA	✓	L	✓	✓
14. Support reasoners	OG	✓	NA	NA	✓	✓	✓

The functions for information retrieval and filtering can be used to implement software for WS discovery and composition, such as Magallanes [21]. Magallanes is a discovery engine that uses MAPI to access information (functionality 1) stored in different repositories. MAPI also makes intensive use of the search for compatible WS with a datatype in the Tool module for the automatic generation of workflows (functionality 5). Magallanes is also available as a plugin for jORCA.

### Use case – homologous protein finder

In this scenario, a bioinformatician has obtained a protein sequence and wishes to know whether this protein has been isolated in another species or even if the protein has any isoform into the same species being studied. The bioinformatician knows the protein identifier and wishes to search in a database for additional information. This example is obviously basic and only involves retrieving the sequence from a database and then comparing that sequence against other known sequences but the purpose of this use case is to illustrate the usage of MAPI functionality (API calls).

### Actors

• WS provider

• Bioinformatician

### Steps

1. The WS provider deploys two BioMOBY WS with the following metadata:

a. Name *getAminoAcidSequence*: input *id* type *Object*, output *sequence* type *AminoAcidSequence*

b. *Name runRPSBlast*: input *sequence* type *GenericSequence*, output *blast_report* type *BLAST-Text*

**Figure 3:**
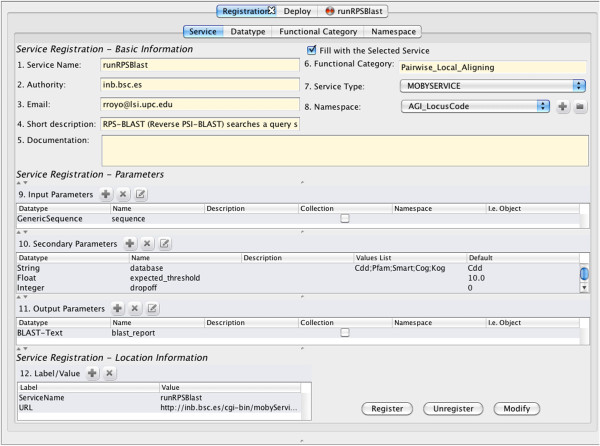
**Registering a service using Flipper. **This shows the metadata necessary for registering the service runRPSBlast. The application Flipper used in this screenshot utilizes MAPI functions to register the service in a BioMOBY service registry.

**Figure 4:**
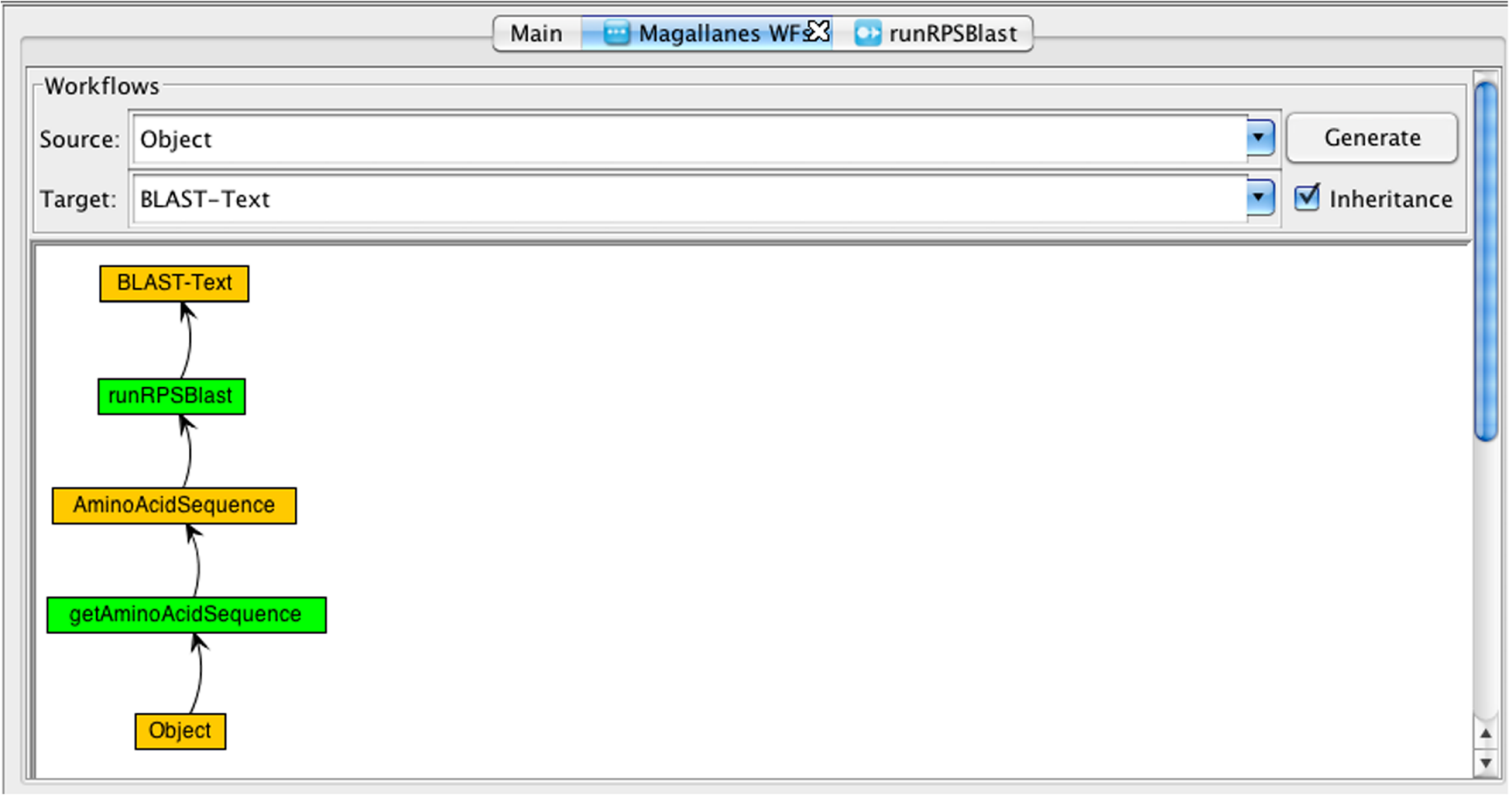
**Discovering a workflow using Magallanes. **This shows how a pipeline can be generated using Magallanes. The application uses MAPI functions to discover the workflow. In many cases, there are several possible paths (compatible services). In those cases, the user can select the most appropriate service (see [17] for details). Please note that MAPI recognizes that the services *getAminoAcidSequence* and *runRPSBlast* can be connected even if the output datatype of *getAminoAcidSequence* is *AminoAcidSequence* and the input datatype of *runRPSBlast* is *GenericSequence*. This is possible because of the inheritance relation between the datatypes (*AminoAcidSequence* ISA *GenericSequence*).

**Figure 5:**
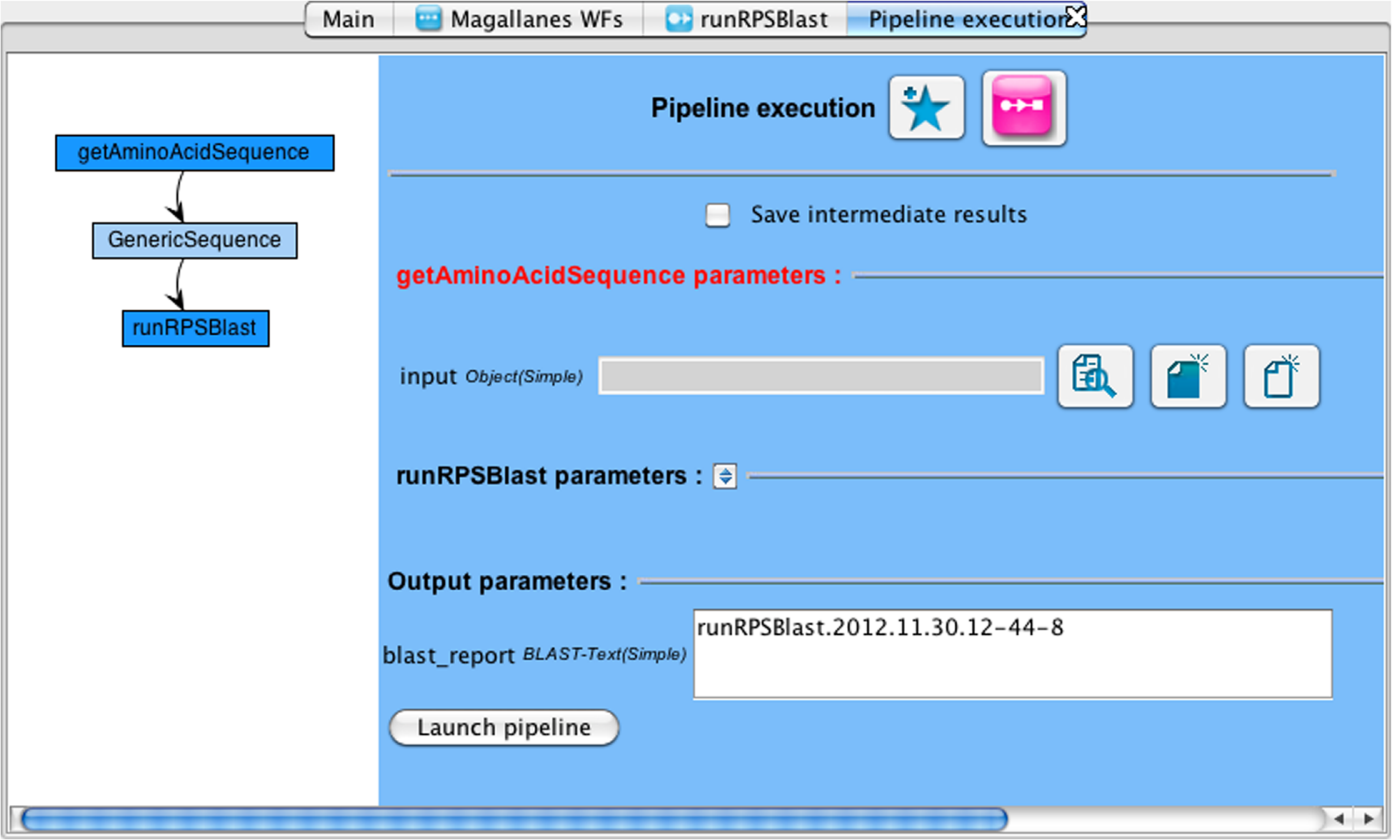
**Enacting a workflow using jORCA. **This shows how the workflow generated in Figure 4 can be enacted using jORCA. The user is required to enter the initial input data for *getAminoAcidSequence* and can, if necessary, modify secondary parameters to fine-tune the enactment.

2. The WS provider registers (see Figure 3) the corresponding WS metadata using the Flipper application [22] which, in turn, uses the following MAPI functions ToolModule:newTool, Tool:addOperation, Tool:addParameter (in that order) to add an abstract definition of the WS and ToolLocationModule:newToolLocation to add specific details related to the protocol (in this case BioMOBY), such as the endpoint where the WS was deployed.

3. The bioinformatician uses the WS client jORCA (please see Section “Clients implemented using MAPI”). She searches for a potential path from input datatype *Object*, output datatype *BLAST-Text*. This is performed by the Magallanes components (see Figure 4) which, using MAPI functions, obtains a representation of the output datatype (DataTypeModule:getDataType), asks the ToolModule which tools produce an instance of this datatype (using calls to ToolModule:getToolList, Tool:getOperations, Operation:getParameters to obtain instances of Parameter), looks at the datatypes of those parameters etc. until it finds an optimum “path” between the requested input and output datatypes. In this case, the datatypes differ slightly (the input datatype of *runRPSBlast* is not the same as the output datatype of *getAminoAcidSequence*). However, since tools in BioMOBY can accept data instances with subtypes of their declared input datatypes, Magallanes can determine that the services are compatible using calls to DataType:isSubtypeOf (*AminoAcidSequence* is a subtype of *GenericSequence*). The datatype ontology from INB is a good example of an ontology that would give good results for this service composition. Obviously this pipeline is very simple (only two services) but a more advanced example, together with details about this procedure, can be found in [21]. More complex service compositions could be imagined for other services and datatypes where the formats differ (in this specific example both services required BioMOBY formatted XML). In more complex cases, MAPI would apply Formatters and Loaders to (if possible) make the data compatible for the next service in the pipeline. Please see the MAPI API documentation and example code snippets in additional files from [9] for further details on this process.

4. Once the tool composition (i.e. pipeline) has been identified, the bioinformatician can enact the pipeline from within jORCA. jORCA knows which input parameters are necessary by using MAPI to obtain the necessary parameters for the first WS using the MAPI function Operation:generateInterface.

5. Once the bioinformatician has provided the necessary input (see Figure 5) and started execution with jORCA, the relevant MAPI worker for the service will be called automatically when calling the ExecutionModule:addTask method.

6. Once running, jORCA will continuously ask the Task object produced by the previous addTask method for the status and, eventually, the results.

This brief example shows the main functionality of MAPI with regards to WS registration, discovery and invocation.

### Dealing with heterogeneity

The main goal of the MAPI framework is to enhance interoperability and compatibility between different technologies by acting as a bridge for their combined use. It is necessary to consider the following aspects in order to combine WS:

1. Syntactic heterogeneity: differences in data formats and service protocols

2. Semantic heterogeneity: differences in the meaning of concepts

MAPI provides metadata which can be used for syntax-based searches (plain-text descriptions of WS functionality) and searches based on semantics (classification of functionalities or WS input/output datatypes). Such datatype metadata can be used to dynamically compose different WS into workflows.

If we combine WS which use and produce data in different formats, we have to take into account several aspects:

• Different names/identifiers, which could be handled in MAPI by adding another Access layer where the identifiers are mapped. For example, most DDBJ [16] service parameters were declared in their WSDL as plain strings. Therefore, we have annotated the parameters with (semantic) datatypes used in the INB service registry in the Access layer implemented for this repository. This enables clients to perform more exact WS discovery (and WS composition).

• Differences in data structure and format, which could be handled by the use of Loaders in MAPI. Loaders are able to modify the structure of a given datatype in order to adapt it to a new structure. For example, as was noted earlier, sequences in BioMOBY contain information about the length of the sequence, whereas standard formats such as FASTA do not. When using the BioMOBY datatype taxonomy in MAPI, sequences in FASTA format are loaded and structured according to the datatype model for sequences (one part with the sequence and another with the calculated length of the sequence). We have several implementations of heuristics based on rules and regular expressions [23], which can recognize biological sequences in different formats. Other heuristics can be specified to recognize further formats like PDB formats, Blast- or ClustalW-outputs. These heuristics allow software clients to recognize raw input data and suggest a reasonable classification to the user. Similar efforts have been undertaken with shim-services in myGrid [24].

### Limitations

In this section, we will discuss limitations in the MAPI approach and give examples of specific solutions.

### Model differences

To add support for new standards or systems, it is necessary to map the new information to be added to the model of the module in question. For example, for mapping WSDL-described WS, it was necessary to map the contents in WSDL to the Tool, ToolLocation and Datatype modules (this task took one person approximately one week to complete these accesses). Note that once the code has been developed, it can be re-used for different services: for example, MAPI uses the same access to obtain metadata about EBI web-services as it does for WABI web-services.

However, in some cases, it is not possible to make a complete mapping from the original source to MAPI. For example, in the data model of MAPI, all WS are considered to have operations but BioMOBY WS do not conceptually provide operations. Therefore, we created a virtual operation in the MAPI representation of BioMOBY WS. So far, we have not encountered major problems related to modelling differences when developing access components for new service types.

### Functionality differences

As can be seen in Table 1, it is difficult to agree on a general set of features for all systems. For example, in the case of BioMOBY, metadata editing is not supported in the API. Therefore, the BioMOBY access provides this functionality by de-registering and registering the metadata instance again with the modified information in a transparent way for the software developer. This method raises the possibility of information inconsistency because the resource can be related with other resources. In this case, de-registering temporarily leaves the repository in an inconsistent state. However, as de-registering and subsequent re-registering is almost immediate, no information inconsistency in the BioMOBY repository has been reported so far.

### Format conversions

MAPI supports plug-ins, which facilitate the interoperation of data in multiple formats. This is possible with many formats, but in some cases the conversion is incomplete since the target format does not support the complete data. For example, a gene sequence can be extracted from a GenBank record and then exported as a BioMOBY object, but the process cannot be reversed since the information discarded from the GenBank record cannot be recovered. This problem is, however, not specific to MAPI and is impossible to avoid because the data formats support different amounts of information.

Format conversion simplifies the integration of heterogeneous WS in many cases, but the approach with loaders/formatters is not possible in all cases. For example, because the Loaders load the full user data into the main memory, huge data sets are not feasible because of memory limitations. However, this depends on the loader implementation. MAPI only provides the interface and does allow extensions of the functionality. For larger data sets, it would be possible to implement a loader which only loads parts of the data “on-demand” into memory and avoids loading the entire dataset at once.

### Future work

In our opinion, WS for biomedical applications must support user authentication, transport encryption, call-by-references and long-running data processing.

Initial work has been started for MAPI to support these requirements. MAPI now support RESTful WS protocol deployed on cloud computing platforms through a new worker implementation [25]. This worker is able to communicate with the WS using user credentials (please see the MAPI tutorial pages for more information about this worker). The worker does not send data directly to the WS but instead sends a data reference which the WS uses to retrieve and process the data. Because some data processing can take considerable time, the communication with the service is split in several steps, submitting the input data references, polling for status and, finally, retrieving the resulting data references. These new developments for MAPI show the flexibility of the suggested architecture: registering service parameters as “data references” and supporting a new WS protocol.

### Alternatives to MAPI

In order to illustrate the coverage of MAPI (in terms of functionality) in comparison with the state-of-the-art frameworks, we evaluated several software frameworks with similar functionalities (see Table 1): BioMOBY, Globus [26], UDDI [27], Feta [28], WSMX [29] and SADI [30]. Many frameworks do not support all functionalities. For example, the API of BioMOBY datatypes only supports querying for the derived datatypes of a certain datatype but not for the parent datatypes (functionality 3). WS can be tagged in standard UDDI systems to indicate functionally, but such tags are not organized hierarchically (functionality 3). It is therefore difficult to use Table 1 as a direct way to compare MAPI with the state-of-the-art and it should instead be used as an indication of the functionality coverage of the frameworks in question.

As we mentioned previously, the BioMOBY framework inspired MAPI. SADI, a recent evolution of BioMOBY, aims to simplify publication and integration of stateless, independent and transformative WS in bioinformatics. SADI advocates a set of best-practices and guidelines which simplify WS composition (chaining). Among those sets of best-practices we can note that SADI WS use HTTP standard operations (for example submitting data is a POST operation and WS descriptions can be obtained via a GET operation). These WS descriptions basically follow the MyGrid/BioMOBY pattern but with references to OWL ontologies for the input/output parameters. The WS descriptions are also available at a public registry which provides a central point for client software during discovery of available SADI WS. SADI WS accept and return RDF messages which are instances of the OWL classes declared in the service descriptions. One side-effect is that SADI WS calls can be made “by reference” simply by letting the instance contain a URI to some external data source.

In many ways, SADI extends the BioMOBY standard; it uses the same service description class as BioMOBY/MyGrid but has moved from using SOAP as a protocol to HTTP standard operations (GET, POST) and to sending RDF data instead of the non-standardized formatting of BioMOBY. Service parameters are annotated with the semantic datatype like in BioMOBY, but are generalized to use any ontology. SADI defines its core metadata according to BioMOBY/MyGrid, which is also the inspiration for the MAPI core metadata set.

It is difficult to compare SADI and MAPI because they fundamentally aim at different things: SADI is a set of best-practice guidelines and reference implementations aiming to simplify publishing semantically well-described services, while MAPI is a software framework for building clients wishing to use different types of WS standards and metadata registries. MAPI is designed to work with any web-service or service metadata registry regardless of the protocol, while SADI recommends a specific protocol.

Another popular tool for computational analysis of genomic data is the Galaxy platform [31]. The aim of Galaxy differs from MAPI; the former provides a web-based workbench for storing and sharing data aimed at end-users (bioinformaticians), while the latter is a framework, which provides a uniform representation of resources available over the Internet, in particular for Web Services. One goal of Galaxy is to make computation accessible for end-users without programming knowledge. MAPI requires programming knowledge and is aimed at software developers developing programs. Much, but not all, of the functionality of Galaxy is provided in MAPI by external software clients such as jORCA and Flipper which both use MAPI. jORCA, for example, also aims to be accessible and useful for non-programmers. Like Galaxy, jORCA presents tools in a standardized interface. In the case of jORCA, this is possible because it uses the standard view of tools in MAPI where all tools are presented the same.

However, a detailed discussion is outside of the scope of this paper. For a more complete summary of the functionality of external software clients for MAPI, we refer to their respective papers (jORCA [20], Magallanes [21]).

In Additional file 2: Supplementary Material (“Comparing WSMX with MAPI”), we discuss the approach taken in WSMX compared to the one of MAPI.

## Conclusions

Internet has boosted the development of numerous resources that are remotely accessible in diverse application domains. For this reason, the need for discovering the right WS for data processing is increasingly urgent and so is the ability to uniformly invoke different WS and combine them to create complex workflows.

In this paper, we have described a framework for developing clients which integrate different resources and systems. This work is an effort toward integrating different tools, repositories and data sources into a unique, flexible and extensible system. To meet these objectives, the system masks the differences in the information structure used by the different resources and provides a uniform representation of such resources to the software developer.

The framework is organized into independent modules which can be combined in different ways. This design is flexible, extensible and proportional. It is flexible in the sense that it can be used to model different types of systems. It is extensible in the sense that new descriptors and even new modules can be developed to provide new functionalities or access new resources. Finally, it is proportional in the sense that developers only need to install those modules which implement the needed functionality.

Every module consists of at least two layers: one layer for reading and writing metadata from their original source (Access) and another layer to expose the data in a uniform way (Interface). The inclusion of new implementations of Access and Interfaces allows the extension to new repositories or new concepts respectively.

Our framework also addresses data heterogeneity (from the point of view of data formats). MAPI attempts to simplify the use of data in different data formats in the following ways:

• A configurable and extensible set of heuristics can be used to recognize the data format of user provided data.

• A configurable and extensible set of formatters/loaders which are able to read a user data file in a given format and access/modify data in a structured way (mapped to the MAPI internal data model as defined in the datatype module).

This is only a partial solution to data heterogeneity and we recognize that this is still an open problem.

As a proof of concept, MAPI provides support for BioMOBY WS, WSDL–described SOAP WS (for example, from European Bioinformatics Institute, EBI and DNA Data Bank of Japan, DDBJ), Taverna workflows, WS from the INB and ACGT projects.

MAPI modules have been successfully used to implement a set of tools targeted at the biomedical domain, a field which uses a large number of formats, protocols and types of tools. These tools range from the simplest, such as a format parser or a file browser, to the more complex, such as a complete tool for the discovery of WS (Magallanes) or a full software suite for the execution of tools (jORCA).

We plan to extend MAPI with access to WS registries such as BioCatalogue [1] and the SADI registry and metadata [30]. Additionally, we are planning to develop a worker component to invoke SADI WS.

WS support machine-to-machine interoperability over a network. However, a weakness of this approach is that WS can differ in their definition, invocation protocols, communication and data formats, preventing service interoperability. MAPI contributes to the ‘high level’ integration of bioinformatics WS by offering a unique model to represent WS and providing the functionality to create client software able to work with different types of WS.

### Availability and requirements

**Project name:** Modular API (MAPI)

**Project home page:**http://www.bitlab-es.com/mapi/

**Operating system(s):** Platform independent

**Programming language:** Java

**Other requirements:** None

**Licence:** MAPI binaries and documentation are under the *Creative Commons Attribution-No Derivative Works 2.5 Spain* license and MAPI source code is available under *GPL v3* license.

**Any restrictions to use by non-academics:** None

## Competing interests

The authors declare that they have no competing interest.

## Authors’ contributions

JK has coordinated the manuscript and the adaption of MAPI for the ACGT project [19]. OT has coordinated and organized the entire development process. Both authors have been involved in the drafting of the manuscript, read and approved the final manuscript.

## Supplementary Material

Additional file 1 **Supplementary Material Internal Data Model. **This document describes in detail the metadata model used in MAPI. Click here for file

Additional file 2 **Supplementary Material Comparing WSMX with MAPI. **This document describes in detail the metadata model used in MAPI and a comparison of WSMX and MAPI. Click here for file
