# Supplementary material for: MAPI: a software framework for distributed biomedical applications
Source: J Biomed Semantics. 2013 Jan 11;4:4. doi: 10.1186/2041-1480-4-4 (PMC3558448; doi:10.1186/2041-1480-4-4)
Supplement: Additional file 1 — Supplementary Material Internal Data Model. This document describes in detail the metadata model used in MAPI. [file 2041-1480-4-4-S1.pdf]

Internal data models

MAPI: a software framework for distributed biomedical applications

Johan Karlsson and Oswaldo Trelles

November 29, 2012

1 Data models

MAPI represents information about web-services and datatypes as follows:

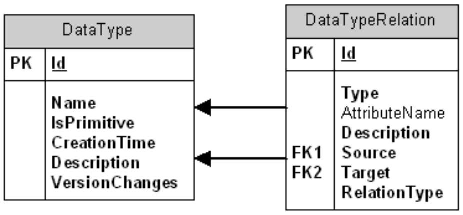

Figure 1: Model for data types. As can be seen, the basic concept is the DataType with name and description etc. Relations between data types (the structure of the taxonomy) are modelled by the DataTypeRelation concept.

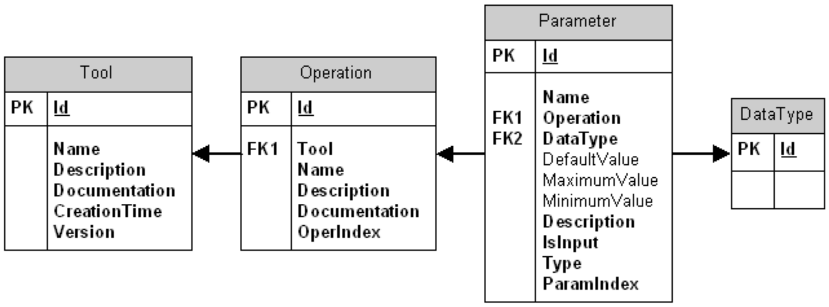

Figure 2: Model for tools. In this case the main concept is Tool, which consists of a set of operations, which, in turn, has a set of parameters. In the case of the parameters, it is possible to represent if the parameter accepts single or multiple values and the maximum or minimum values. Finally, we can note that the relationship between this module and the data type module is established through the operation parameters which indicate the type of data for the input/output parameters.

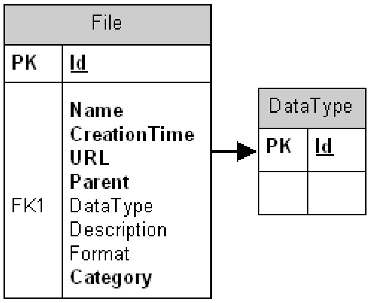

Figure 3: Model for files. As shown in the figure, the model consists of a single table to model two concepts (files and directories). The field category is used to differentiate between these types. For both resources, we can specify attributes such as name or date of creation, and most importantly, the directory where the file or directory is located, with the attribute *Parent*.

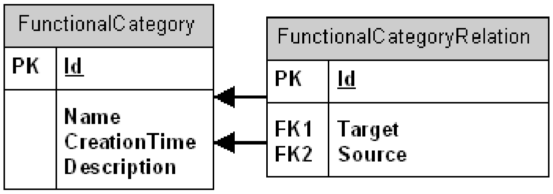

Figure 4: Model for functional categories. This module is responsible for providing a mechanism to define functional categories which organize the different types of resources. Each category has a name, description and date of creation. It is also possible to establish relationships with other categories to organize them hierarchically. Each category can have several parents.

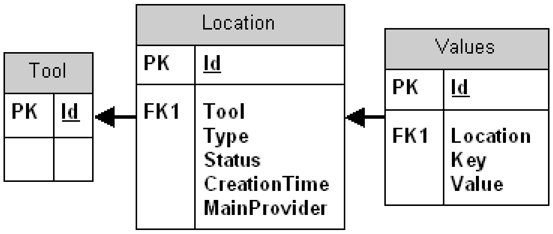

Figure 5: Model for locations. This module is focused on the management of service instances and therefore the data model has been oriented to allow the management of the diverse information in these resources. The information is organized in two main parts: one generic part with information about service instances (the table *Location*); with information such as type of tool (for example, BioMOBY service) or the creation date of the metadata. In the table *Values* it is possible to specify all values needed to invoke a specific tool. Because it is impossible to predict this (depends on the invocation protocol), the information in this table are stored as tuples of the type *key / value*.

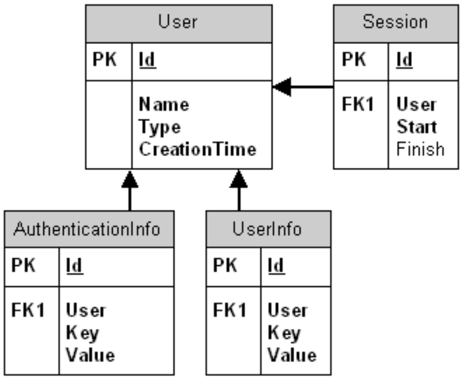

Figure 6: Model for users. Information about users is very diverse, so we have used the same solution as for locations. In this case the information has been divided into general information and information used for user identification. The model also includes information about the user creation date and type (Administrator, Registered or Temporary) and the ability to control the sessions that the user has opened and their duration.

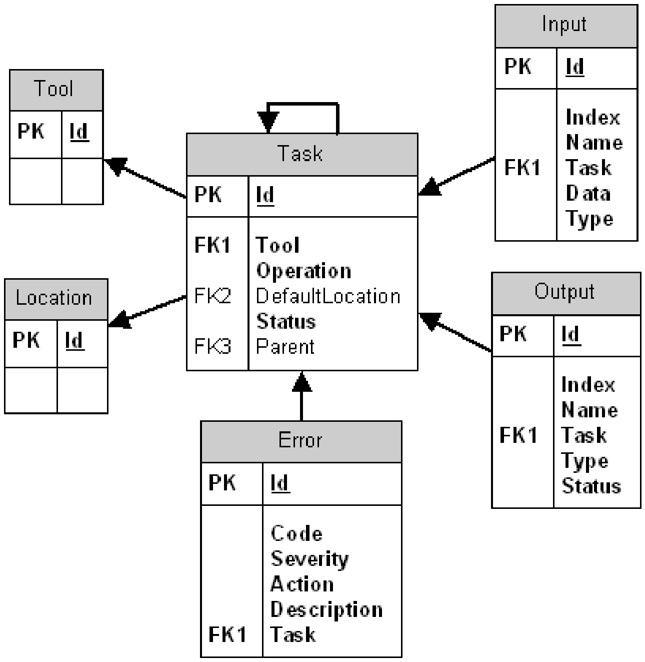

Figure 7: Model for the run time (tasks). The main focus in this model is the tasks, representing the processes running. The tasks indicate the tool and operation invoked and the current state of the task. Tasks can produce error messages which are represented in the error table, with error code, a description of the error occurred, the severity (*Error* - total failure or *Warning* - partial results produced).

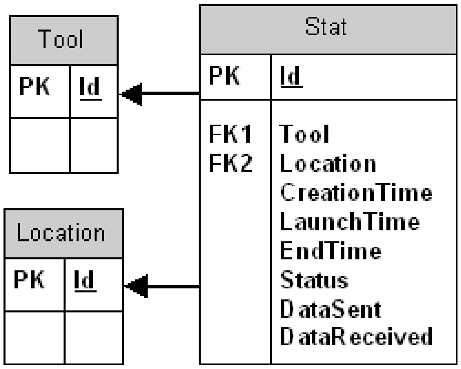

Figure 8: Model for statistics. This module will model the statistics produced by the execution of different tasks. The model represents which tool was invoked, which location, time of task creation/launching and completion. It is also possible to represent the size of data sent and returned.

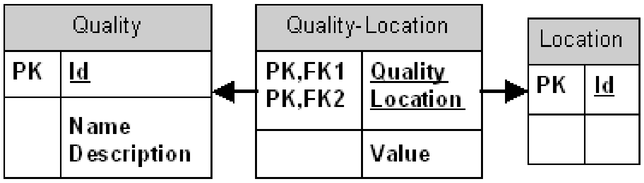

Figure 9: Model for tool location qualities. This model can extend the information in the model for location with information about features such as asynchronous calls supported, error control or data persistence mechanism. This consists of a feature name and a brief description.

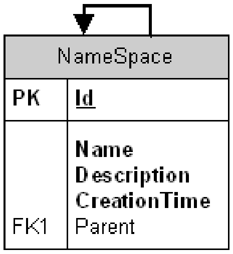

Figure 10: Model for namespaces. This model represents information such as the name, description and creation time of namespaces. It is also possible to associate namespaces with each other for hierarchical organization.
