# Supplementary material for: MAPI: a software framework for distributed biomedical applications
Source: J Biomed Semantics. 2013 Jan 11;4:4. doi: 10.1186/2041-1480-4-4 (PMC3558448; doi:10.1186/2041-1480-4-4)
Supplement: Additional file 2 — Supplementary Material Comparing WSMX with MAPI. This document describes in detail the metadata model used in MAPI and a comparison of WSMX and MAPI. [file 2041-1480-4-4-S2.pdf]

Supplementary Material

Comparing WSMX with MAPI

Johan Karlsson and Oswaldo Trelles

November 30, 2012

1 Discussion

In this supplementary material, we will compare MAPI with WSMX which provides a very similar set of functionalities but uses a different approach. For example, WSMX uses the Pellet semantic reasoner to filter resources in searches and MAPI provides a mechanism where Java classes implement the filtering mechanism directly. In our opinion, this is easier for typical software developers to understand because most basic training in informatics covers Java programming. In a similar way, MAPI is a more open system with respect to the technology and standards for WS, for example WSMX currently only supports SOAP WS described in WSDL. Support for other types of WS, such as CORBA, must be made through a WSDL binding.

1.1 Filtering

Compared to the WSMX approach with support for standardized reasoning using, for example, the pellet reasoner, the mAPI approach with constructing filters using programmatic subrutines (in Java) is more flexible and since most computer science education programmes include Java programming, it is likely easier for the typical software developer to write filters in Java.

1.2 Service focus

WSMX currently only supports SOAP services described using WSDL. The semantic service descriptions are however generic and there are plans to also support REST-style services. There are WSDL bindings for CORBA services. There is support in MAPI for several kinds of services, in particular for BioMOBY style services and R-scripts.

1.2.1 Scheduling

There is no explicit support for scheduling in WSMX. These kinds of tasks would likely be handled by individual services which internally could call to a computational grid for solving given tasks. Data about service availability and cost is available and used for service selection but there is no proper scheduling. The MAPI has explicitly been designed to support scheduling. This will be realized in the execution module which can select different mirrors depending on service statistics (average replytime etc.). Currently the scheduling algorithm is being developed but the overall design explicitly supports this.

1.3 WSMX service discovery

The user specifies an abstract goal which contains information about what the user wants to accomplish. During web service discovery, the abstract goal is matched with the abstract definitions of the services (stored in repositories) in order to provide a solution that achieves the given goal. The static descriptions of the services are complemented with other non-functional information during the service discovery, for example, how often the service is used in the given context, how often the service is available etc.

If suitable services are found, the user is presented with these services and performs validation of the proposed services. If the user agreed, the system selects a concrete service among the suitable services, based on non-functional information. At this stage, the actual communication with the service is started according to the specific service protocol semantics.

If the abstract service descriptions are specified with different ontologies, data mediation is performed based on mapping rules between the involved ontologies. Such mapping rules are created during registration of the service in a repository and are needed to permit data mediation.

The service discovery and validation stage are potentially time-consuming and the system therefore stores goals and suitable services in the repository for later retrieval and presentation to the user.
